# Heterogeneous Information Fusion for Robot-Based Automated Monitoring of Bearings in Harsh Environments via Ensemble of Classifiers with Dynamic Weighted Voting

**DOI:** 10.3390/s25175512

**Published:** 2025-09-04

**Authors:** Mohammad Siami, Przemysław Dąbek, Hamid Shiri, Anna Michalak, Jacek Wodecki, Tomasz Barszcz, Radosław Zimroz

**Affiliations:** 1 Faculty of Geoengineering, Mining and Geology, Wrocław University of Science and Technology, Na Grobli 15, 50-421 Wrocław, Poland; 2 School of Electronics and Computer Science (ECS), University of Southampton, Southampton SO17 1BJ, UK; 3 Faculty of Mechanical Engineering and Robotics, AGH University of Kraków, Al. Mickiewicza 30, 30-059 Kraków, Poland

**Keywords:** condition monitoring, bearing monitoring, CNN, information fusion, dynamic voting, transfer learning

## Abstract

Modern inspection mobile robots can carry multiple sensors that can provide opportunities to take advantage of the fusion of information obtained from different sensors. In real-world condition monitoring, harsh environmental conditions can significantly affect the sensor’s accuracy. To address this issue in this paper, we introduced a fusion approach around information gaps to handle the portion of false information that can be captured by the employed sensors. To test our idea, we looked at various types of data, such as sounds, color images, and infrared images taken by a mobile robot inspecting a mining site to check the condition of the belt conveyor idlers. The RGB images are used to classify the rotating idlers as stuck ones (late-stage faults); on the other hand, the acoustic signals are employed to identify early-stage faults. In this work, the cyclostationary analysis approach is employed to process the captured acoustic data to visualize the bearing fault signature in the form of Cyclic Spectral Coherence. Since convolutional neural networks (CNNs) and their transfer learning (TL) forms are popular approaches for performing classification tasks, a comparison study of eight CNN-TL models was conducted to find the best models to classify different fault signatures in captured RGB images and acquired Cyclic Spectral Coherence. Finally, to combine the collected information, we suggest a method called dynamic weighted majority voting, where each model’s importance is regularly adjusted for each sample based on the surface temperature of the idler taken from IR images. We demonstrate that our method of combining information from multiple classifiers can work better than using just one sensor for monitoring conditions in real-world situations.

## 1. Introduction

In recent decades, different mobile robotic platforms have been developed to perform inspection tasks in hazardous industries. Human–robot collaboration in the mining industry is considered a solution to improve human safety and production quality [1,2,3]. Monitoring the condition of critical industrial infrastructure is considered a vital task in reducing the possibility of sudden breakdowns in production lines. Therefore, to ensure production safety, the machines involved in the process must be inspected in a timely manner [4,5,6,7,8].

Bearings are considered an integral part of every rotating element, an important example being the idlers used on belt conveyors (BCs), which on mining sites are the logistic machinery responsible for the transport of the mining production [9,10,11]. The average length of a mining BC can be counted in kilometers, with thousands of idlers that need to be monitored to ensure the safety of the production line [12].

The mobile robot can carry multiple sensors that can be intelligently used to monitor rotating machines in harsh and difficult-to-reach environments for humans [13,14,15]. Non-contact instruments are considered proper tools to assess the machine’s condition, as they can reduce the complexity of measurement through robot-based inspection tasks. Mobile robots might be capable of carrying various non-contact instruments, such as laser scanners based on light detection and ranging (LiDAR), laser Doppler vibrometers, high-speed cameras, microphones, or IR cameras. However, considering harsh conditions in mining sites, laser Doppler vibrometers and high-speed cameras can be ineffective considering factors such as uneven surfaces (unstable movement of the robot) or low ambient light. However, IR and RGB images, together with the acoustic samples collected by a microphone, could be more robust to environmental noise in a harsh environment.

Although single-sensor measurements are complementary to the condition monitoring of rotating machines, to accomplish more complex tasks, multi-sensor configurations are becoming increasingly important. The processing of data collected in a multi-sensor configuration is considered an extremely complex task, as the availability of heterogeneous data leads to the need to develop fusion methods that are compatible with the complexity of the monitored equipment [16,17].

The continuous development of deep learning methods has received attention due to their strong nonlinear feature extraction performance. However, due to the limitation of the extraction of multiple features in single-modal samples, improving deep learning approaches could be limited to the novelty of the information in the extracted samples. Therefore, to improve the performance of models driven by deep learning in fault diagnosis, heterogeneous information fusion approaches have received the interest of researchers.

In our proposed condition monitoring approach, an inspection mobile robot equipped with three different cameras, including RGB and IR imaging cameras, and a microphone is used to capture heterogeneous sources of data that can be used to identify different fault stages of damaged idlers. We individually analyze the advantages of each data source while mentioning their limitations.

Information fusion approaches might be separated into two categories, including feature-based fusion and decision-based fusion. The feature-based fusion is more suitable for problems where the fused information (homogeneous data) is captured by sensors of the same type. However, in problems where the dimensions of captured data are different due to sensor properties, the fault information (heterogeneous data) properties cannot be easily recognized due to the different characteristics and distribution of the features studied. Therefore, decision-level fusion methods can be used to make deep learning-based classifiers more accurate in identifying bearing faults.

Ensemble learning refers to learning approaches that combine several baseline models—in our case deep learning-based classifiers—to take advantage of fused information from individual classifiers. It can be used to build a single large model that is more accurate than every individual classifier [18,19,20]. In this work, we implemented a dynamic weighted average voting fusion approach to fuse the decisions of the CNN models employed for classification of the pre-processed RGB images and acoustic signals captured from idlers.

A thermal anomaly on the idler surface should be considered a sign of a serious defect in the idler bearing. To improve the accuracy of the fusion results, we defined the idler surface temperature for each of the measured idlers. The normalized value of the idler temperature in each case with respect to the temperature of other inspected idlers is used to dynamically set the weights in the employed voting method to improve the accuracy of the proposed fusion approach.

In this paper, we investigate the use of an inspection mobile robot for condition monitoring of BC idlers and present a practical, multi-sensor data-processing pipeline that exploits heterogeneous, robot-acquired inputs—RGB and infrared (IR) images together with acoustic recordings—to detect and stage idler faults. The principal technical contribution is a systematic evaluation of TL variants of classical CNN backbones as sample-efficient feature extractors on real, in-field robot data; we demonstrate that TL-based feature extraction, paired with conventional machine learning classifiers, provides robust classification performance under the constraints of limited, imbalanced field datasets. The experimental comparison comprises eight deep models derived from four CNN backbones, and two widely used classifiers—Random Forest (RF) and XGBoost—applied to the extracted features. Compared with end-to-end multimodal deep architectures, our approach emphasizes operational practicality and annotation efficiency for inspection robots, while enabling straightforward decision-level fusion of visual, thermal, and acoustic modalities. The general contributions of the paper are summarized below:

- We apply cyclostationary analysis to robot-recorded acoustic signals (e.g., cyclic spectral coherence) to extract fault-specific features that are robust to the non-Gaussian, high-interference noise typical of in-field mining environments, enabling earlier detection of bearing/roller defects.
- We propose a dynamic, temperature-aware weighted voting fusion scheme in which per-sample classifier weights are adaptively adjusted using normalized idler surface temperature from IR imagery; this improves sensitivity to thermally driven fault stages.
- We demonstrate that transfer learning-based feature extraction combined with classical classifiers (Random Forest, XGBoost) and the proposed fusion strategy yields accurate and stable classification performance on highly imbalanced, small-sample datasets.
- We validate the approach on BC datasets acquired in operational mining sites, reporting real-case performance and conducting experiments to quantify the contribution of each modality and the fusion rule.

## 2. Literature Review

Information fusion is a fundamental process that involves combining observations or information from multiple different sources to provide a robust, complete, or more precise understanding of an environment or process of interest [21,22]. The goal is to achieve a fuller or more accurate description of reality than can be gained by considering individual sources separately.

Most current data fusion methods employ probabilistic descriptions of observations and processes, often using Bayes’ Rule to combine information. In mathematical terms, Bayes’ theorem can be described as the probability of a hypothesis conditional on a given body of data, to the “inverse” probability of the data conditional on the hypothesis [23].

Bayes’ Rule is central to most data fusion methods, enabling inferences about an object or environment (described by a state) given an observation. For multi-sensor inference, Bayes’ Rule requires conditional independence of observations and results in the posterior probability being proportional to the product of the prior probability and individual likelihoods from each information source. The recursive form of Bayes’ Rule is advantageous as it only requires the storage and computation of the posterior density, which summarizes all past information, allowing for sequential updates as new observations arrive [22].

Basic probabilistic modeling and fusion techniques include Grid-based models [24,25] and Kalman Filters [26,27]. The Information Filter, a dual of the Kalman filter, is noted for its relative simplicity in the update stage, especially for systems with multiple sensors, as it translates products of likelihoods (from Bayes’ Rule) into sums. This property is exploited in robotic networks and navigation problems [28].

Beyond the basic probabilistic approaches, there exists a wide array of different methodologies. The reputation-based approach in generalized and unified form has been discussed in [29], where the authors used the beta reputation system based on the Bayesian formulation. The approach there is explained from the transactional point of view, where two cooperating nodes (such as sensors) exchange information (data from measurement). Each exchange generates a “cooperativeness rating”, which will later be used to determine the probability of future exchanges between the nodes. Similar algorithms have found their use in the recent rise of multi-agent systems relevant in artificial intelligence advancements. Examples are Distributed Reputation Mechanism [30], Deep Reinforcement Learning-based reputation model with Multi-Agent Deep Deterministic Policy Gradient [31] or information search applications [32].

Metric-based fusion operates on the concept of similarity (through metrics such as distance or correlation) between observations from different sensors for proper data integration. The difference in value of chosen metric allows one to define the trust in individual sensors (reduced upon large value disagreement). This approach is especially effective for data measured in common feature space, such as fusion of LiDAR and camera sensors [33,34] or multiple sensors of the same type [35].

The authors of [36] discussed applications of the Dempster–Shaffer evidence theory in multi-source data fusion. This method and its further generalizations (e.g., Dezert–Smarandache Theory) in contrast to most other approaches work under uncertainty and can deal with conflicting information. Techniques such as fuzzy logic-enhanced Kalman filters are another option to deal with this problem, noticeably used in robot localization and positioning problems [37,38,39].

The voting approach as proposed in this article has already been successfully used in sensor fusion applications. Examples can be an axlebox bearing fault diagnosis, where fusion is used to merge multichannel data information into the final result [40] or in the fusion of SAR images with optical sensor data [41]. The voting methodologies are still being improved, such as in [42], where the authors implemented universal generating function, or in [43], where the authors proposed a dual weighted voting algorithm for K-nearest neighbor classification.

Recent developments in sensor technologies have enabled researchers to propose new fusion-based condition monitoring methodologies to identify faults in rotating machines with higher precision [44,45,46,47,48]. While traditional vibration-based analysis remains prevalent, different sensors, including acoustic, infrared (IR), RGB cameras, and current, offer complementary insights into equipment health. However, the fusion of multimodal data presents challenges due to differences in sampling rates, signal resolutions, and environmental susceptibility. For example, acoustic emission (AE) sensors excel in capturing high-frequency stress waves generated by incipient faults such as micro-cracks or lubrication failures [1]. However, their efficacy diminishes in noisy environments such as mining sites, where mechanical collisions could corrupt signal integrity. In contrast, non-contact IR thermography and RGB imaging provide robust visual indicators of overheating or surface defects in harsh settings but lack sensitivity to early-stage faults, as they primarily detect thermally or visually manifested anomalies [49].

To address these challenges, deep learning architectures—particularly convolutional neural networks (CNNs)—have emerged as powerful tools for fusing heterogeneous data streams. Gültekin et al. [45] pioneered a deep residual network (DRN)-based fusion framework to diagnose bearing faults under variable load and speed conditions. Their method converts raw vibration and current signals from six synchronized sensors into time–frequency representations via the short-time Fourier transform (STFT), enabling the DRN to learn cross-sensor spectral patterns. Similarly, Kou et al. [46] fused vibration, motor current, and IR images for CNC machine tool wear monitoring. They employed Gramian angular difference fields (GADFs) [47] to encode 1D time-series data into 2D texture images, preserving temporal correlations. A hybrid CNN processed these alongside the IR images.

Despite progress, critical gaps persist. First, most studies evaluate fusion models under controlled laboratory conditions, neglecting real-world constraints such as sensor misalignment, intermittent data loss, and variable sampling rates. Second, considering the fact that CNN models need to have access to be successfully trained makes them challenging models to choose, as, in industrial settings, it could be rather expensive and, in some case studies, impossible to acquire enough samples for training the models. Although techniques like transfer learning [48] and synthetic data generation are proposed as remedies, their efficacy in multi-sensor fusion contexts lacks rigorous validation. Lastly, as long as different variations of CNN architectures have been rapidly developed in the past decade, their performance evaluation in different sources of data is necessary, which is merely studied at present. In this direction, the practicality of the deep learning approach needs to be measured when it comes to training the models on a limited number of samples, as a known major drawback of a CNN model is the requirement for a large amount of training data.

## 3. Material and Methods

**Figure 1:**
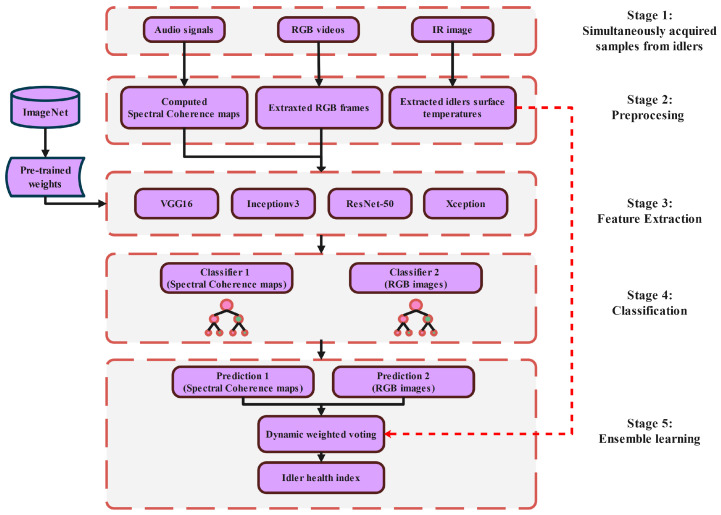
Simplified flowchart of the proposed fusion-based diagnostic method.

In this work, we propose a robotics-based approach for the automation of belt conveyor idler monitoring at mining sites. The mobile inspection robot in our work has collected different sources of data, including acoustic signals, IR, and RGB images from BC idlers in real-world scenarios. The simplified flow diagram of the proposed data fusion approach is shown in (see Figure 1).

The proposed methodology consists of five stages, including the acquisition and pre-processing stage, where data acquired by the inspection mobile robot are first stored and undergo various pre-processing methods to increase the chance of identifying the fault pattern. In the third phase, we used CNN architectures as feature extractors. Moreover, we study the application of two different feature classification methods, namely, RF and XGBoost. In the fourth phase, a dynamic weighted voting ensemble-based approach was considered to fuse the classifiers’ decisions to make a final prediction. The main idea of this voting approach is that the chance of facing classification errors in individual classifiers can be reduced by merging particular decisions through a dynamic weighted average voting scheme. Finally, we demonstrate the overall performance of the proposed data fusion approach in comparison to single-sensor measurement methods.

### 3.1. Cyclic Spectral Coherence

In the analysis of rotating machinery, the identification of modulation frequencies is essential for different carrier frequencies. To address this, the cyclic spectral analysis is introduced. Antoni [50] introduced cyclic spectral coherence (CSC) to quantify this phenomenon. Let us begin by recalling the cyclic power spectrum (CPS) $S_{X}\left(f,\alpha \right)$ of the signal x:

(1) $$S_{X}\left(f,\alpha \right)=\lim_{L\to \infty }\frac{1}{L}E\left(F_{x,L}\left(f+\frac{\alpha }{2}\right)\bar{F_{x,L}\left(f-\frac{\alpha }{2}\right)}\right),$$

where $F_{x,L}\left(f\right)$ is the Fourier transform of the signal x calculated over an interval of length *L*; $\alpha =[\alpha_{1},\ldots ,\alpha_{A}]$ is the modulating frequency; and $f=[f_{1},\ldots ,f_{F}]$ is the carrier frequency. According to Equation (1), CPS measures the dependence of the spectral components spaced by a given modulation frequency α for a given carrier frequency *f*. The cyclostationary signal should show $\left|S_{X}\left(f,\alpha \right)\right|>0$ for some modulation frequency α≠0. Based on the CPS definition, the formula for SC is introduced as follows [50]:

(2) $${\left|\gamma_{X}\left(f,\alpha \right)\right|}^{2}=\frac{{\left|S_{X}\left(f,\alpha \right)\right|}^{2}}{S_{X}\left(f+\frac{\alpha }{2},0\right)S_{X}\left(f-\frac{\alpha }{2},0\right)}$$

This normalized statistic, within the interval (0,1), quantifies the spectral cyclic autocorrelation of the signal. It serves as an indicator of cyclostationarity. A value close to one implies a cyclostationarity property of the signal at the carrier frequency (*f*) with a modulation period of T=1/α. The estimation of SC, as per Equation (2), can be performed directly using the CPS estimator. Specifically, the estimator of CSC is given by

(3) $$CSC\left(f,\alpha \right)={\left|{\hat{\gamma }}_{X}\left(f,\alpha \right)\right|}^{2}=\frac{{\left|{\hat{S}}_{X}\left(f,\alpha \right)\right|}^{2}}{{\hat{S}}_{X}\left(f+\frac{\alpha }{2},0\right){\hat{S}}_{X}\left(f-\frac{\alpha }{2},0\right)}$$

Here, ${\hat{S}}_{X}\left(f,\alpha \right)$ is an estimator of the CPS, with various methods presented and compared in [50]. In this article, the Welch method is applied.

In this study, we utilized acoustical analysis to investigate the operational condition of idlers, integral components of belt conveyor systems utilized for the transportation of bulk materials in the mining industry. The acoustic signals emanating from these idlers were captured using a mobile robot, resulting in a dataset composed of recordings from 17 distinct idlers. The acquired signals exhibit a temporal extent of 6 s, sampled at a frequency of 48 kHz.

**Figure 2:**
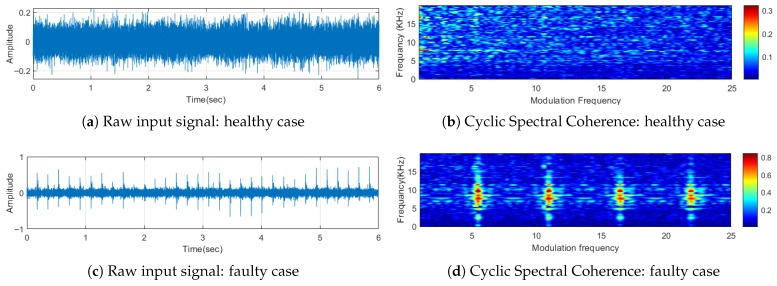
Comparison of the common energy patterns in Cyclic Spectral Coherence for a faulty and healthy idler.

**Figure 3:**
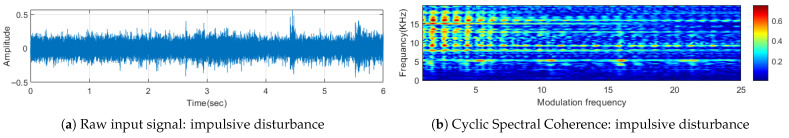
Time–frequency representation of the signal with impulsive disturbance due to belt joint using a metal clip.

**Figure 4:**
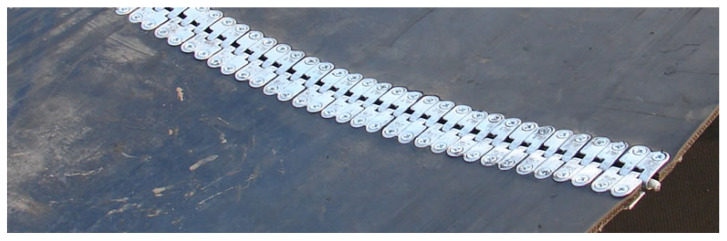
Belt joint using a metal clip.

Illustrated in Figure 2 are representative examples that feature a healthy idler, a faulty idler, and a scenario involving the influence of a conveyor belt joint. The panels Figure 2a and Figure 2b present the raw signal and the corresponding CSC map of the pristine idlers, respectively, showcasing the baseline acoustical profile. In contrast, panels Figure 2c,d depict the raw signal and the Cyclic Spectral Coherence of faulty idlers, thereby highlighting deviations from the norm. Furthermore, in Figure 3, the panels Figure 3a,b offer information on the raw signal and the Cyclic Spectral Coherence associated with the sound emanating from the conveyor belt joint during signal acquisition (see Figure 4). This is an important example to show that although other noise sources can show cyclic behavior, it will have a different characteristic.

This comprehensive analysis allows for a nuanced understanding of acoustic characteristics, facilitating the identification and differentiation of healthy and defective idlers, as well as discerning the impact of conveyor belt joints on the audio profile.

### 3.2. RGB Image Pre-Processing

During the examination, the inspection mobile captured continuous RGB videos of the wing idlers that were located at the top of the BC. The healthy idler must rotate continuously to move the belt along the conveyor. In some severe cases, the bearings in the idlers can be damaged, resulting in a sudden stop in idler rotation. The failed idlers can be recognized in the IR camera due to heat generated due to friction between the idler and the belt. However, because of the absence of rotation, the fault cannot be recognized in the captured acoustic signal; therefore, it is essential to analyze the health status of the idlers using the RGB images.

**Figure 5:**
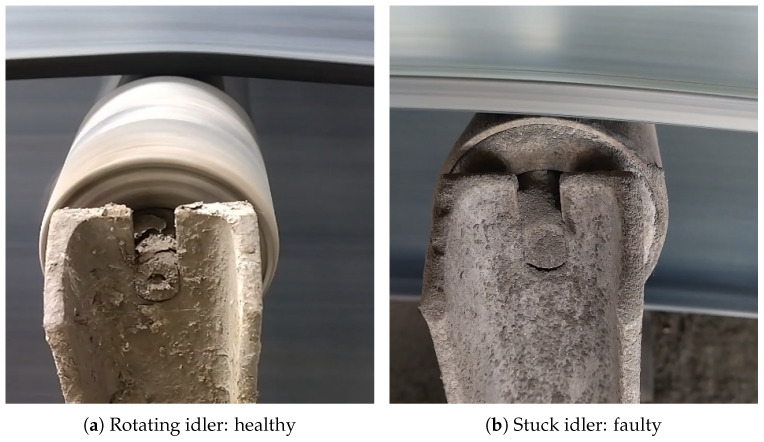
Comparison of RGB images captured from a moving and stuck idler.

Initially, an input video is divided into separate frames $Frames=\left[fr_{1},fr_{2},fr_{3},\ldots ,fr_{n}\right]$ where each frame must be processed and classified individually. The original size of the frames extracted from the raw video file were (720 × 720 × 3) pixels, which is too large to be processed using the CNN models employed. Therefore, the size of the frame sequence is reduced to (256 × 256 × 3) pixels for training and testing the CNN models. In Figure 5, we demonstrate the two idlers to compare the difference between the rotating idler in pre-processed RGB frames.

### 3.3. IR Image Processing

**Figure 6:**
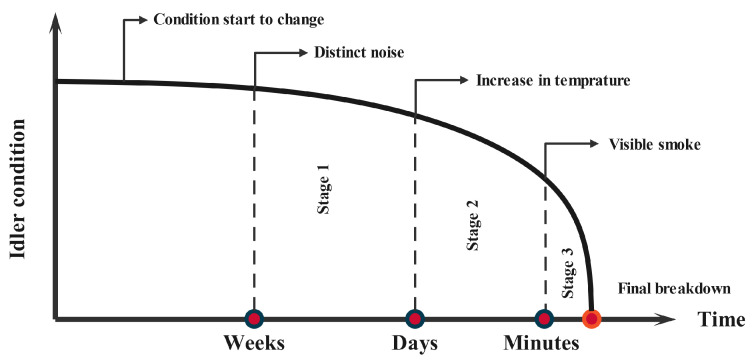
Evolution of failure signals in belt conveyor idlers.

In Figure 6, we show the evolutionary signs of the faults on conveyor belt idlers due to damage over time. It can be seen that there is a continuous relationship between the fault signature in different stages of development. Moreover, one can notice that temperature changes on idler bearings can be detected when the idler condition is close to failure; therefore, anomalies in the idler surface should be considered as an important measure to identify faulty idlers.

In previous sections, we discussed the advantages of RGB images and acoustic signals in the diagnosis of idler bearings. Both measures are important tools for identifying the fault at its early and late stages; however, the IR image as an efficient tool can give us additional robust information in a time frame that the supervisor would have enough time to replace the faulty idler.

In this study, to improve the overall performance of the proposed information fusion scheme, we extracted the idler surface temperature from the examined idler using the IR camera and introduced the normalized value as a weight in the dynamic weighted voting approach.

**Figure 7:**
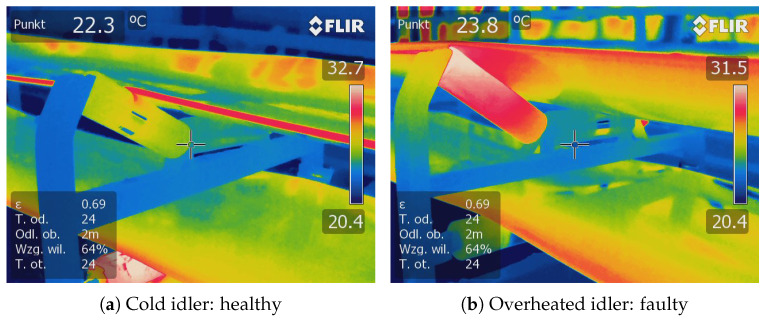
Comparison of IR image from a cold (moving idler) and overheated (stuck idler).

In Figure 7, we demonstrate the IR image captured from the idler represented in Figure 5. As can be seen, friction between the stuck idler and the moving belt generates huge heat that could be captured by the IR camera carried by the inspection robot.

### 3.4. Data Description and Augmentation

Through this research, we studied the different sources of data, including acoustic signals and RGB and IR images captured from 17 different idlers using a mobile robot. After initial data pre-processing, we noticed that only 4 out of 17 monitored idlers were faulty. Two acoustic samples indicate early-stage faults (idler numbers 12 and 13); however, we did not capture temperature anomalies on the surfaces of diagnosed idlers with early-stage faults. The reason was that since the faults did not fully develop in the idler bearings, there was no sign of a thermal anomaly on the idler surfaces. On the other hand, we notice two stuck idlers (final stage faults) using the captured RGB image with signs of thermal anomalies (idlers numbers 15 and 17).

These numbers can indicate that our original dataset suffers from the class imbalance problem, which can significantly affect the performance of the semantic segmentation model in the correct detection of overheated idlers. In this way, training deep learning classifier models can become a crucial issue [51].

Oversampling and undersampling are the most common techniques for addressing model overfitting and class imbalance issues. Data augmentation can be considered an oversampling method to amplify minority classes [52,53]. In this direction, we employed a different approach for oversampling the pre-processed RGB images and acoustic signals.

#### 3.4.1. RGB Image Augmentation

In our case study, the inspection mobile robot passed along with each idler and collected the RGB image samples from different angles of 17 different idlers. To create a balanced dataset, we increased the number of positive samples (stuck idlers). We selected 15 samples captured from different angles of faulty idlers while selecting 2 samples from healthy idlers. In doing so, we created a base dataset with 60 samples that includes an equal number of positive and negative samples.

In addition, we employed different image augmentation techniques to increase the number of samples to train the classifiers in the next step. The image augmentation techniques can be divided into three different categories: geometric and color space transformations and pixel point operations. In this work, different data augmentation techniques, namely vertical flip, random rotation at 90 degrees, horizontal flip, and transpositions, have been applied to RGB image datasets. It is worth mentioning that image augmentation techniques have been applied to datasets using the Albumentations package [54]. After augmentation, we created a dataset that included 240 samples with an equal number of positive and negative samples to train and test the classifiers.

#### 3.4.2. CSC Map Augmentation

To increase the set of CSC maps obtained from positive (faulty) samples, a two-step augmentation was proposed:

- Step 1: In this step, the speckle noise is added to the CSC map, which is usually modeled as the multiplicative noise (Rayleigh noise). The degraded data point in a CSC map (denoted as $I(f_{i},\alpha_{j})$) can be defined as follows:
  (4) $$I\left(f_{i},\alpha_{j}\right)=CSC\left(f_{i},\alpha_{j}\right)\cdot N\left(f_{i},\alpha_{j}\right),\;f_{i}\in f,\alpha_{j}\in \alpha$$
  where $CSC(f_{i},\alpha_{j})$ is the original CSC map in point $\left(f_{i},\alpha_{j}\right)$ and $N(f_{i},\alpha_{j})$ represents the multiplicative Gaussian noise with mean equal to 1 and standard deviation equal to 0.05.
- Step 2: The noisy map is then convolved with a 2D Gaussian kernel that is used to blur images. A 2D Gaussian smoothing kernel is applied to the samples (x,y) using the following equation:
  (5) $$Z\left(x,y\right)=\frac{1}{2\pi \sigma^{2}}e^{-\frac{x^{2}+y^{2}}{2\sigma^{2}}},$$
  where σ is sampled uniformly from the interval [0,1] for every augmented instance. This controlled blurring reduces artifacts while introducing additional intra-class variability.

The sequential application of added noise, followed by scale-randomized smoothing, produces realistic yet distinct CSC realizations, enhancing class balance and improving model generalization in downstream fault detection tasks.

Spectral correlation maps were assigned to three categories: faulty, belt joint (external disturbance), and healthy. The original corpus comprised 17 maps in total (2 faulty, 4 external-disturbance, and 11 healthy samples). To reduce the pronounced class imbalance, samples in the underrepresented classes (faulty and belt joint) were synthetically expanded using the augmentation procedures described previously. After augmentation, the dataset was expanded to 35 examples and adjusted to achieve near-balanced representation across the three classes. This augmented, more balanced set was then used for training and evaluation to mitigate bias towards the majority class and to improve the classifiers’ ability to learn discriminative spectral features for rare fault conditions.

### 3.5. Feature Extraction

A CNN is a type of neural network that is designed based on the cognitive mechanism of the biological visual system [55]. CNN-based approaches are widely regarded as the most popular methods in the field of graphics processing due to their strong performance in image processing and the ability to directly handle raw images. CNN uses convolution filters, pooling, and other operations to extract image features. The model is trained using gradient descent and back propagation algorithms to perform tasks, including image classification [56]. CNN architecture typically consists of five layers: the input layer, the convolution layer, the activation layer, the pooling layer, and the fully connected layer. In the following, we briefly explain each layer:

- **Input layer**
  This is the access point for unprocessed image data. Within this layer, images can undergo pre-processing through various operations, such as normalization, principal component analysis, and whitening. Pre-processing standardizes images, which could accelerate the training of network models and consequently enhance model performance.
- **Convolution Layer**
  The primary layer of a CNN is responsible for performing convolutions on the input images in order to extract relevant visual features. In general, a convolution layer consists of multiple convolution kernels, which act as filters to extract various features of the image.
- **Activation Layer**
  The purpose of this layer is to apply a nonlinear mapping to the convolution results, allowing the multilayer network to exhibit nonlinearity and enhance its expressive capacity. The rectified linear unit (ReLU) function and the Sigmoid function are frequently employed as activation functions.
- **Pooling Layer**
  This layer is commonly referred to as the down-sampling layer. Its purpose is to reduce the dimensionality of the extracted features and compress the data. This helps to mitigate overfitting and enhance the model’s fault tolerance. Pooling techniques encompass MaxPooling and AveragePooling, with MaxPooling widely used at present.
- **Fully Connected Layer**
  This layer serves as the output layer and is responsible for achieving the function of classifying objects. The function of this layer is to consolidate the feature information obtained from each individual neuron in the layer above and subsequently categorize the images according to the desired outcome.

#### Classical CNN Architectures and TL

Some of the CNN architectures proposed in the past decade have received considerable attention due to their exceptional performance in performing different image processing tasks. The advantages of CNN in image recognition are revealed annually in the ImageNet Large-Scale Visual Recognition competition [57]. In this work, we select the four classical CNN architectures to perform feature extraction on pre-processed RGB images and acoustic samples.

TL enhances the performance of CNN by leveraging pre-existing knowledge from a source domain into a target domain. This enables the CNN model to increase its pattern recognition capabilities or handle new tasks in limited labeled data. Empirical evidence has demonstrated that CNN-TL variants exhibit a commendable generalization. Furthermore, compared to prototype-based approaches, CNN-TL models showcase a more robust ability to extract patterns beyond the scope of the training data.

In this paper, the used model was initially trained on the ImageNet dataset. The ImageNet dataset contains more than 13 million pictures from 20,000 categories, allowing the network to be deeply trained on a different range of images [58]. The training weights were used to perform feature extraction on our dataset. In the following, we briefly describe the key advantages of each employed model:

- **VGGNet16**
  In VGG16 architecture, there are 13 convolutional layers, five Max Pooling layers, and three dense layers. It was first introduced by Oxford University and Google DeepMind first introduced it in 2014 to improve the AlexNet architecture by replacing large filters with sequences of smaller 3 × 3 filters [59].
- **ResNet 50**
  The Residual Network (ResNet) architectures take advantage of the concept of skip connections, which allow the network to learn deeper representations without overfitting. There are multiple versions of the ResNet architecture with different numbers of layers. In this article, we use ResNet50, first introduced in 2015 [60]. It consists of 48 convolutional layers, one MaxPool layer, and one average pool layer.
- **InceptionV3**
  The Inception architecture was first developed by Szegedy et al., who changed the straight-up and straight-down serial network to the parallel sparse connection network [61]. Furthermore, the researchers used the global average pooling layer to replace the fully connected layer. In this paper, we employed the inceptionv3 variation to perform feature extraction tasks. Inceptionv3 was designed to allow the use of deeper networks, while also controlling the growth of parameters.
- **Xception**
  As an improvement over Inception, the Xception was first introduced in 2017 and uses the depth-wise separable convolution layer to improve the convolution layer within InceptionV3 [62]. Xception is a CNN that is 71 layers deep, has fewer parameters and, therefore, is faster than Inception.

### 3.6. Feature Classification

Generally, at the latest stage of a CNN architecture, a fully connected layer is responsible for performing the feature classification task. In our work, we switched out the fully connected layers for XGBoost and RF models. The proposed approach based on CNN fusion with RF and XGBboost has been studied to see how the extracted features in the convolutional layer can be used to classify input samples—in our case, pre-processed RGB images—and spectral coherence maps into the desired classes. To perform this, the classifier models have been separately trained using the features extracted from the training dataset. Subsequently, the test dataset was used to measure the final performance of the proposed approach in performing a true classification.

### 3.7. Random Forest

The Random Forest algorithm is a type of supervised learning. Creates a “forest” by combining many decision trees that are trained using the “bagging” method. The fundamental principle of the bagging approach is that the aggregation of multiple learning models enhances the final result. Furthermore, it can be implemented by creating multiple decision trees during training, and the output can be obtained by averaging the predictions of each unique tree [63].

### 3.8. XGboost

XGBoost utilizes randomization approaches, such as random subsamples and column subsampling, to minimize training time and reduce the risk of overfitting. By employing a compressed, presorted columnar data storage system, the computational expense of finding the best split can be reduced. Utilizing a columnar storage structure allows for concurrent examination of the most efficient partitioning of each attribute being assessed. XGBoost employs an approach that does not involve scanning all possible candidate splits. Instead, it uses data percentiles to evaluate a reduced subset of probable splits and determine their benefit utilizing aggregated statistics. Thus, the implementation of this notion has been achieved through the process of subsampling data at the node level [64].

### 3.9. Ensemble Learning

Classification models play the main role in identifying the fault pattern in the acquired data, as we previously discussed. The accuracy of the CNN models is highly dependent on the dimension and type of dataset. As a single source of data cannot be used to identify faults in their different stages, fusion algorithms have been employed in this article to improve the overall performance of the introduced bearing diagnosis framework.

The ensemble model can be generated by combining the base models to develop a robust one. The ensemble model can employ CNN models with different architectures to solve a classification problem that cannot be easily addressed by either of the individual models. In this work, we used an ensemble learning approach using dynamic weighted voting to fuse the decisions of the employed models that were separately trained and tested on pre-processed coherence maps and RGB images.

In the ensemble learning approach, we consider the output of each classifier as input to the fusion models. Generally, ensemble frameworks can be defined based on two characteristics. The first characteristic can be defined as the trained baseline models, whether they are sequential or parallel [65,66]. The second can be defined as the fusion method, which is the selected approach to combine the output of the baseline classifiers using different voting approaches.

#### Dynamic Weighted Voting Method

Ensemble learning systems generally rely on an aggregation function G(·) that combines the outputs of *h* base classifiers $c_{1},c_{2},\ldots ,c_{h}$ to predict a single output. The dataset can be defined as

(6) $$D=\left\{\left(x_{i},y_{i}\right)\right\},\;1⩽i⩽n,$$

where $x_{i}\in R^{m}$ is a feature vector of dimension *m* which represents the sample after pre-processing and feature extraction, *n* is the size of the dataset, and $y_{i}$ is the corresponding label. The prediction of the output based on this ensemble method can be defined as [67]:

(7) $$y_{i}=\phi \left(x_{i}\right)=G\left(c_{1},c_{2},\ldots ,c_{h}\right)$$

In this paper, we utilize the concept of the parallel ensemble technique [65], where decisions are generated simultaneously, as there is no data dependency. Therefore, each classifier was trained using a different source of data, including a pre-processed RGB image and an acoustic signal. The main reason for this is that it leverages the independence between the base learners. Therefore, the errors generated by one classifier differ from those found in another independent classifier, allowing the ensemble model to calculate the average errors [68].

To integrate the outputs of the baseline classifiers into a single output, we employed a dynamic weighted average voting approach. The voting method can be used in classification problems to improve predictive performance. The idea of averaging voting is that the predictions are extracted from multiple different classifiers, and an average of the predictions is used to make the final prediction. The main limitation of average voting is that it is assumed that all baseline models are equally effective. The average prediction can be computed using arithmetic mean, which is the sum of the predictions divided by the total predictions, as describe below:

(8) $$y^{*}=\underset{k}{\mathrm{argmax}}\frac{1}{h}\sum_{l=1}^{h}w_{kl},$$

where $w_{kl}\in \left[0,1\right]$ is the probability $k^{th}$ class label of the $l^{th}$ classifier [67].

The weighted average voting method is a slightly modified version of averaging voting where different weights are given to the baseline classifiers to indicate the degree of importance of each model in prediction. There are two main methods of weighting that can be identified: dynamic weighting and static weighting of classifiers [69]. During the operational phase, the dynamic method allows the weights assigned to individual classifiers to vary for each input vector. In the static technique, weights are calculated for each classifier during the training phase and remain constant during the classification of input patterns.

The dynamic weighted average voting method is better in terms of accuracy compared to the simple average voting method. The difficulty in employing a weighted average ensemble lies in determining the correct weight for each classifier. Furthermore, the computation involved in this method is more complex due to the need to calculate the weighted average of the prediction results from all baseline models.

As shown in Figure 6, this study employs three complementary sensors: a microphone to capture audio signals, a thermal camera to measure idler surface temperature, and an RGB camera to monitor idler rotation and visible damage. The RGB camera is used primarily to detect gross mechanical failures (e.g., severely damaged or seized idlers) by identifying the absence of rotation or other clear visual defects.

**Figure 8:**
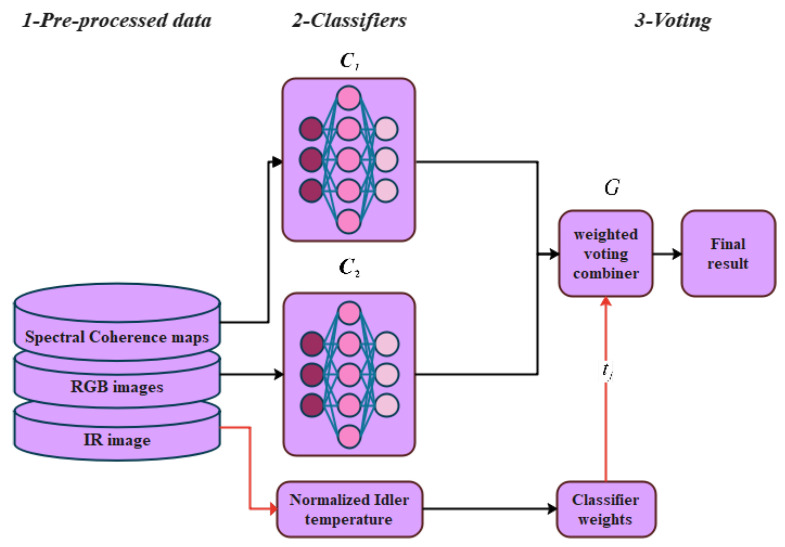
Simplified flowchart of the proposed dynamic weighting method.

To produce a balanced decision-making scheme that takes advantage of information from all sensors, we propose a dynamic weighting function based on normalized idler surface temperature. This approach allows the system to recognize cases in which severe damage produces a non-rotating idler, and therefore, no anomaly appears in the captured audio. Because the audio signal classifier is most effective in detecting early-stage faults in rotating idlers, it is assigned a constant weight of 1 so its importance remains unchanged across samples. The classifier responsible for detecting late-stage faults from RGB images receives a temperature-dependent weight: when thermal evidence indicates an elevated risk, the RGB contribution is increased, improving detection of severe damage, particularly in cases where distinguishing rotating from stalled idlers from visual data alone is challenging (see Figure 8).

For a dataset of size *n*, each input sample has an associated temperature value. In this direction, the value of $t_{i}$, which is the weight of the $c_{2}$ classifier, is defined based on the normalized value of the idler temperature studied in the input sample. Normalization ensures that the weight falls within the range [0,1], and is calculated as

(9) $$t_{i}=\frac{t_{input_{i}}-t_{\min}}{t_{\max}-t_{\min}},$$

where $t_{input_{i}}$ is the temperature of the idler in the $i^{th}$ input sample, and $t_{\min},t_{\max}$ are the minimum and maximum observed idler temperatures in the dataset, respectively.

The RGB-based classifier produces a continuous score in the interval [0, 1], where values closer to 1 indicate a higher probability that the idler is experiencing a faulty rotation (i.e., mechanically compromised or seized). Similarly, the dynamic temperature coefficient is normalized to the same range, with 1 corresponding to the hottest idler in the dataset. Because stalled or severely damaged idlers tend to produce elevated frictional heating, their temperature coefficients and the RGB classifier scores are both biased toward higher values.

Leveraging the temperature coefficient as a multiplicative or gating factor therefore increases the likelihood of correctly identifying stuck idlers: When thermal evidence is strong (a coefficient closer to 1), the contribution of a high RGB score is amplified, improving true positive detection of severe, non-rotating faults. Conversely, when the temperature coefficient is low, the influence of ambiguous visual evidence is reduced, which helps avoid false positives from visual artifacts. This joint interpretation of the normalized classifier score and the temperature coefficient thus improves detection reliability by aligning the fusion decision with physically meaningful thermal evidence.

Given this temperature-derived weight, the fusion of the two baseline classifiers (i.e., $c_{1}$, $c_{2}$) is achieved using a weighted function, denoted by $G(c_{1},c_{2},t)$, defined as

(10) $$G(c_{1},c_{2},t)=\frac{\sum_{i=1}^{n}\left(c_{2}t_{i}\right)+\left(c_{1}\right)}{1+\sum_{i=1}^{n}t_{i}},$$

where $t_{i}$ is the weight applied to the RGB classifier’s output based on the temperature corresponding to the $i^{th}$ input sample, and $c_{1}$ and $c_{2}$ refer to the CSC map and RGB image classifiers, respectively.

## 4. Performance Metrics

For the evaluation of the proposed classifier, we calculated the following performance metrics: sensitivity, precision, accuracy, and the F1 score.

(11) $$\;\mathrm{Accuracy}\;=\frac{\left(TP+TN\right)}{\left(TP+FN)+(FP+TN\right)}$$

(12) $$\;\mathrm{Sensitivity}\;=\frac{\left(TP\right)}{\left(TP+FN\right)}$$

(13) $$\;\mathrm{Precision}\;=\frac{\left(TP\right)}{\left(TP+FP\right)}$$

(14) $$\;F1\;\mathrm{Score}\;=\frac{\left(2\cdot Precision\cdot Sensitivity\right)}{\left(Precision+Sensitivity\right)}$$

Here, precision is the proportion of correctly classified overheated idlers among the entire population. Sensitivity is measured as the proportion of true positive cases that are correctly predicted by the classifier, while specificity is the prediction of true negative cases that are correctly predicted. Precision is the proportion of correct predictions in the confusion matrix of all positive predictions. Furthermore, the F1 score is the harmonic mean of precision and sensitivity. The coefficient takes into account the factors TP (true positive), TN (true negative), FP (false positive), and FN (false negative) to score the model. The ideal value of these metrics is 1 and it is the target for the models in this study.

## 5. Data Collection

**Figure 9:**
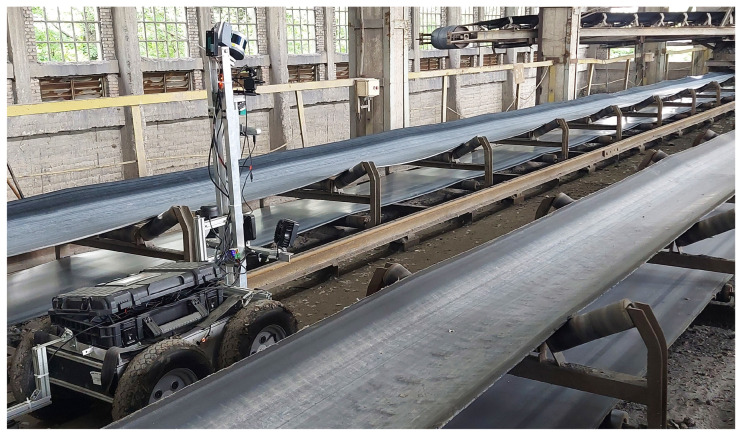
View of the robot during inspection.

In this study, we used a set of data from various sources, such as acoustic signals, RGB images, and IR images taken by a mobile robot at an open-pit mining site, to check the condition of conveyor belt idlers (see Figure 9). The open-pit mining site in this study is located in Jaroszów, 50 km west of Wroclaw. The length of the parts inspected in the conveyor systems was 150 m, where there was a space of 1.45 m between each idler. The detailed description of the mobile robot employed in this research is described in our previous work [49,70].

Through the inspection, the mobile robot moved along the belt conveyor system and captured continuous thermal and RGB images from the wing idlers located on the upper side of the conveyor belt. It is worth mentioning that all the videos were captured from the left side of the studied belt conveyor system. Furthermore, IR videos were captured using a FLIR T640 camera (Wilsonville, OR, USA) with a 45-degree field of view. The format of the captured videos was 768 × 584 pixels, 16-bit-colored videos. The RGB camera with a resolution of 1920 × 584 pixels was used to capture RGB images from idlers as well.

**Figure 10:**
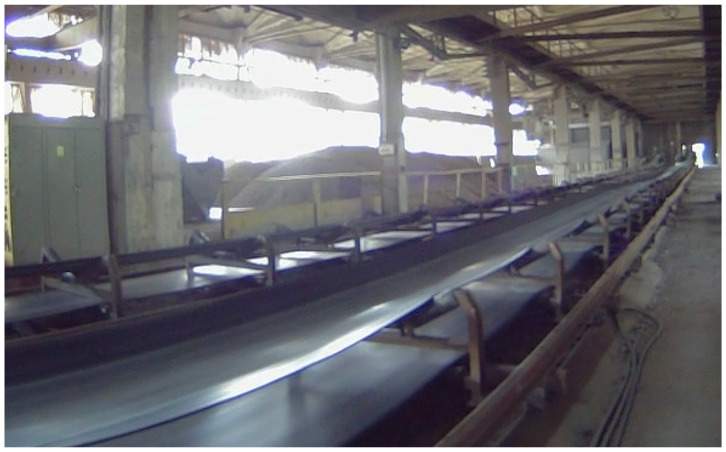
General view of the raw material storage area, showing the belt conveyor used for material transport.

A total of 100 idlers were inspected during the field campaign (see Figure 10). Of these, four idlers were identified as faulty and were selected, together with thirteen healthy idlers, to form the dataset used for classifier development (seventeen idlers in total). The four faulty cases represent 4% of the inspected population, reflecting the low prevalence of failures in real operational conveyor systems. To construct a balanced training set suitable for supervised learning, we intentionally selected this subset for model training. Acoustic data were obtained from the RGB camera’s onboard microphone: six-second audio clips were extracted from the recorded video at a sampling rate of 48 kHz for each sample, yielding acoustic records corresponding to the 17 selected idlers.

## 6. Training Process

As discussed previously, different sources of data captured from 17 idlers were studied. We used balanced datasets to train and test the classifiers from a single-sensor monitoring perspective. As long as different test sets were used to initially train the classifiers, we selected nine synchronized data points from nine idlers to study the performance of the proposed fusion method.

The hardware environment used in this study included the following: an AMD Ryzen 5800H (Santa Clara, CA, USA), an NVIDIA GTX 3060 Ti GPU (Santa Clara, CA, USA), and 16 GB of RAM. The software environment includes the following: Windows 10 OS, Python 3.6, Keras 2.2.4, and Tensorflow-gpu1.12.0. Based on the time complexity of our models using training and validation datasets, we carefully set the experimental parameters of XGBoost and RF to balance the resources used while achieving good performance. The values and meanings of the selected hyperparameters for the RF and XGBoost methods are presented in Table 1.

## 7. Results and Discussion

The trained deep learning models were tested to understand the usability and working performance of the models. Here, the performance factors used are accuracy, precision, and F1 score. An F1 score above 0.9 indicates the usability of the model in real-world applications. The performance of the model using the test dataset is shown in Table 2.

The VGG16 architecture, used as a feature extractor with XGboost as a classifier, achieved the highest F1 score (0.9333) for accurately classifying captured RGB into two clusters: rotating idlers (healthy) and stuck idlers (faulty). The ResNet-50 architecture with RF as a classifier also reached the qualified level of testing performance (0.90). On the other hand, the Xception architecture with RF as the classifier has the lowest F1-test score (0.5882) of the rotating/stuck idler classification.

For the classification of acquired Cyclic Spectral Coherence, we first define three different classes, as we discussed earlier. The performance of two Inceptionv3 and Xception architectures with RF as a classifier was the highest among the studied models in the true classification of Cyclic Spectral Coherence with the F1 score (1). However, the F1 scores of the other models studied were below 0.90, which indicates their unsatisfactory performance in the true classification of optical coherence.

**Figure 11:**
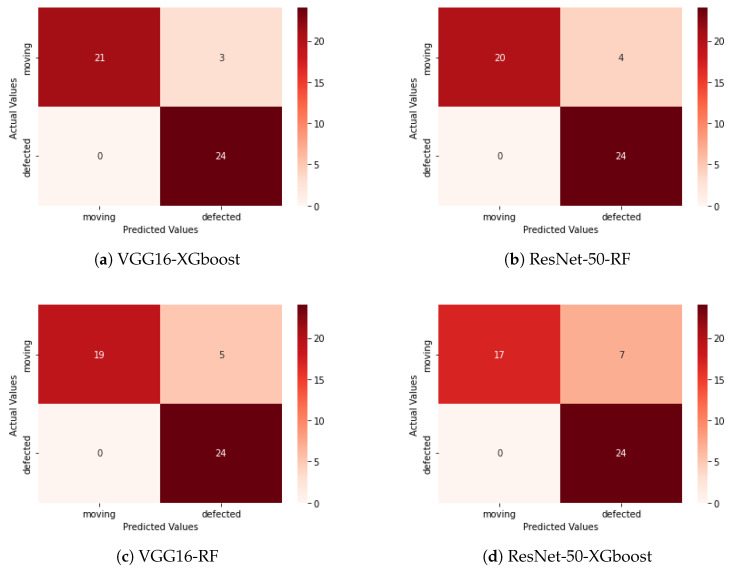
Comparison of confusion matrix of 4 models with the highest F1 score on the classification of RGB images.

**Figure 12:**
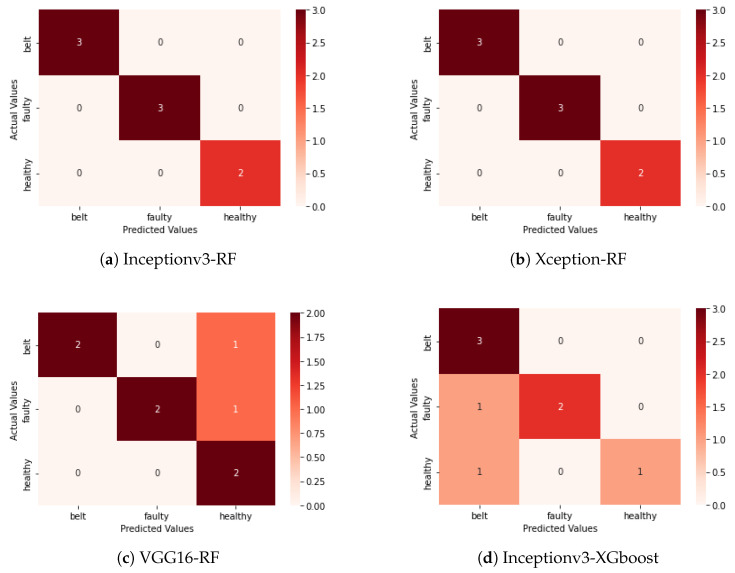
Comparison of confusion matrix of 4 models with the highest F1 score on the classification of Cyclic Spectral Coherence.

In Figure 11 and Figure 12, we show that the confusion matrix selected four of the best models with the highest F1 score. The ordinate axis of the confusion matrix represents the actual label of each class, and the horizontal axis represents the predicted label.

Figure 11 highlights a recurrent failure mode across the evaluated models: permanently stuck idlers are often misclassified as healthy. This deficiency is principally attributable to the scarcity of representative stuck-idler examples in our training set (only 240 augmented stalled-idler samples for training and validation), which constrains the models’ ability to learn robust, discriminative visual features for this class. Nevertheless, the proposed hybrid strategy—using pre-trained convolutional networks as feature extractors, combined with fine-tuned machine learning classifiers (e.g., Random Forest, XGBoost)—delivered strong overall performance, as reported in Table 2. This fusion paradigm is therefore particularly attractive for real-world industrial condition-monitoring pipelines, where labeled fault data are limited and computationally efficient, and generalizable solutions are required.

In operational mining environments, permanently damaged idlers are typically removed and replaced immediately because the sustained belt–idler friction they produce can create a serious fire hazard; consequently, collecting large numbers of real stuck-idler cases in the field is challenging. To mitigate this limitation, we introduce a dynamic weighted voting scheme that incorporates the normalized surface temperature of each idler as an auxiliary weighting factor on the RGB classifier output. Because stalled idlers generally exhibit elevated surface temperatures due to frictional heating, the temperature weight increases the influence of high RGB scores for suspected stuck cases, thus reducing false negatives. The effectiveness of this temperature-modulated fusion is demonstrated later in this section.

The Cyclic Spectral Coherence (CSC) maps exhibited well-separated cluster structures in the studied dataset, enabling the classifiers to discriminate the three target classes with relatively high accuracy. Accordingly, the results in Figure 12 and Table 2 show that classification performance on CSC inputs exceeded that obtained on RGB images; this improvement is attributable to the lower intrinsic complexity and clearer class-specific patterns present in the CSC representations.

Nevertheless, this finding should be interpreted with caution because the CSC experiments relied on a very limited training corpus (35 augmented maps), which reduces statistical confidence and may overstate generalization performance. To mitigate data scarcity, we exploited convolutional backbones pre-trained on large-scale natural-image datasets (ImageNet) for feature extraction; these pre-trained models effectively transfer to CSC inputs because the maps have simpler, lower-dimensional structure than typical RGB scenes, enabling robust feature encoding even with few labeled examples. The downstream classifiers (Random Forest and XGBoost) trained on these high-quality deep features proved sample-efficient and delivered strong results, illustrating that classical machine learning classifiers can perform well when supplied with informative, pre-extracted representations.

The performance of individual classifiers demonstrates that automated, robot-based inspection can effectively substitute manual idler condition monitoring under field conditions. However, each sensing modality possesses distinct failure modes and information gaps, so relying on any single model limits reliability. To address this, we adopt a decision-level ensemble strategy: the best-performing base classifier (selected by validation F1) is promoted as a strong expert, and its outputs are incorporated as inputs to the fusion stage (together with the other classifiers and the normalized IR temperature). This ensemble-based refinement leverages complementary strengths across modalities, reduces modality-specific false negatives, and produces a more robust detection model for idler diagnosis.

To compare the performance of the base classifier with the proposed ensemble learning (data fusion model), we redefine the prediction indicator with respect to the actual state of the idler. Therefore, TP indicates the faulty idler, whether the fault is in an early or late stage, while TN indicates a healthy idler.

As shown in Table 3, combining Inceptionv3-RF (Cyclic Spectral Coherence classifier) and VGG16-Xgboost (RGB image classifier) using the normalized temperature of idlers (captured from IR images) as an additional weight to reduce the number of FN in the RGB image classifier results in a lower misclassification rate than individual classifiers. Our approach can accurately identify those faulty idlers that, due to bearing permanent damage, cannot be rotated, and hence they are identified in captured acoustic signals.

## 8. Conclusions

Early detection and precise localization of overheated idlers are essential to prevent unplanned shutdowns in BC systems. The offline workflow discussed in this work enables the use of computationally intensive pre-processing and deep learning models on control-room servers rather than onboard the robot, facilitating more sophisticated analysis without increasing the robot’s payload or power budget. Moreover, by decoupling data acquisition and heavy computation, the robot can resume inspection tasks immediately while the analysis proceeds in parallel, improving operational throughput.

The experiment was carried out during the standard workflow of the facility; therefore, any existing malfunction was not critical to its functionality. Although any other malfunction other than idler-related malfunctions was not taken into consideration (actively looked for), most operational problems would have some sort of reflection in the malfunction occurrence on the idlers, such as some of them not moving, becoming excessively hot, or exhibiting noise. All these faults can be detected through this method—by detecting some malfunction rather than finding the direct cause—which should be further investigated after the faulty idler detection. It is important to note that this article is focused on the faulty idler detection rather than the classification of the problem.

In this work, we developed and validated a multimodal, robot-based condition-monitoring framework for conveyor belt idlers deployed in a mining environment. The system combines acoustic recordings processed using cyclostationary analysis (Cyclic Spectral Coherence), RGB image classification of rotating versus stuck idlers, and IR thermography to extract idler surface temperature. Feature extraction was performed using transfer learning variants of classical CNNs, and the extracted features were classified with RF and XGBoost; final decisions were produced by a dynamic weighted voting ensemble in which the RGB branch weight is modulated by the normalized IR temperature, while the acoustic branch retains a constant prior. Experimental evaluation of field data demonstrates that the temperature-aware late-fusion ensemble reduces false negatives from single-sensor classifiers and improves overall F1 performance compared to individual modalities.

The manuscript’s principal contributions are as follows: Firstly, we apply cyclostationary analysis (CSC) to a mobile-robot’s acoustic data collected under harsh, non-laboratory mining conditions, which improves the visualization and detectability of early fault signatures. Secondly, we introduce a practical dynamic weighted majority-voting fusion rule that adapts classifier weights on a per-sample basis using normalized idler surface temperature from IR images, thereby grounding fusion decisions in physically meaningful thermal evidence. Third, we conduct a comparative evaluation of multiple CNN transfer learning architectures combined with RF and XGBoost classifiers to assess their relative effectiveness within the proposed fusion pipeline. Collectively, these contributions deliver a pragmatic multi-sensor fusion strategy that addresses real-world information gaps and class imbalance typical of robotic inspection scenarios, especially for condition-monitoring idlers in conveyor systems located in mining sites.

This study demonstrates the feasibility of using an inspection mobile robot for condition monitoring of idlers in belt conveyor (BC) systems. While the proposed pipeline shows clear operational advantages, several limitations remain and will be addressed in future work. First, our evaluation is based on a relatively small, site-specific dataset with severe class imbalance; although transfer learning and data augmentation reduce some effects, the limited sample size restricts statistical generalization and necessitates extensive balancing procedures. Second, we evaluated only cyclic spectral coherence for acoustic pre-processing; comparative assessments against other time–frequency and signal-processing techniques are needed to determine the most informative representations for early fault detection. Third, deploying sensors on a mobile platform introduces operational challenge occlusions, variable viewing geometry, intermittent data loss, and high environmental noise that can degrade modality fidelity and hamper out-of-the-box transferability to other sites. To strengthen and extend these findings, we plan to collect larger, multi-site datasets (different mines, conveyor designs, and operating regimes), perform cross-site validation, and investigate robustness measures such as sensor calibration protocols, domain adaptation, redundancy, and missing-modality handling. Implementing these extensions will improve reliability, enhance generalization, and provide stronger evidence of the method’s practical utility for industrial deployments.

## Figures and Tables

**Table 1:** Hyperparameters of the employed RF and XGBoost methods and their values.

Models	Hyperparameters	Meaning	Values
RF	$n_{tree}$	Number of trees used in the forest	50
	$m_{try}$	Number of random variables used in each tree	42
XGBoost	Learning rate	Shrinkage coefficient of each tree	0.3
	Maximum tree depth	Maximum depth of a tree	6
	Subsample ratio	Subsample ratio of training samples	1
	Column subsample ratio	Subsample ratio of columns for tree construction	1
	Maximum delta step	Maximum depth of a tree	0
	Gamma	Minimum loss reduction required to make a further partition	0

**Table 2:** Results of the studied deep learning model on the classification of extracted RGB images and Cyclic Spectral Coherences.

Model	Depth	Number of Parameters	Accuracy		Precision		F1 Score	
			RGB Image	Acoustic Signal	RGB Image	Acoustic Signal	RGB Image	Acoustic Signal
VGG16-RF	16	138.4 M	0.8958	0.75	0.7916	0.7777	0.8837	0.7555
VGG16-XGboost			0.9375	0.75	0.8750	0.7222	0.9333	0.7301
Inceptionv3-RF	189	23.9 M	0.7083	1	0.4166	1	0.5882	1
Inceptionv3-XGboost			0.7916	0.75	0.5833	0.7222	0.7368	0.7388
ResNet-50-RF	107	25.6 M	0.9166	0.625	0.8333	0.6666	0.9090	0.6238
ResNet-50-XGboost			0.8541	0.50	0.7083	0.5555	0.8292	0.4330
Xception-RF	81	22.9 M	0.7708	1	0.5416	1	0.7027	1
Xception-XGboost			0.7291	0.875	0.50	0.8888	0.6486	0.8666

**Table 3:** Performance comparison of proposed sensor fusion in true detection method of damaged idlers.

Source of Information	Accuracy	Precision	F1 Score
Acoustic signal (Inceptionv3-RF)	0.75	0.6	0.75
RGB image (VGG16-XGboost)	0.5	0.2	0.33
Fusion (Acoustic, IR image, RGB image)	0.85	0.80	0.88

## Data Availability

Archived datasets cannot be accessed publicly according to the NDA agreement signed by the authors.

## References

[1] Shiri H., Wodecki J., Ziȩtek B., Zimroz R. (2021). Inspection robotic UGV platform and the procedure for an acoustic signal-based fault detection in belt conveyor idler. Energies.

[2] Dabek P., Szrek J., Zimroz R., Wodecki J. (2022). An Automatic Procedure for Overheated Idler Detection in Belt Conveyors Using Fusion of Infrared and RGB Images Acquired during UGV Robot Inspection. Energies.

[3] Siami M., Barszcz T., Wodecki J., Zimroz R. (2024). Semantic segmentation of thermal defects in belt conveyor idlers using thermal image augmentation and U-Net-based convolutional neural networks. Sci. Rep..

[4] Boloz L., Bialy W. (2020). Automation and Robotization of Underground Mining in Poland. Appl. Sci..

[5] Błażej R., Kirjanów A., Kozłowski T. (2014). A high resolution system for automatic diagnosing the condition of the core of conveyor belts with steel cords. Diagnostyka.

[6] Dąbek P., Krot P., Wodecki J., Zimroz P., Szrek J., Zimroz R. (2022). Measurement of idlers rotation speed in belt conveyors based on image data analysis for diagnostic purposes. Measurement.

[7] Bortnowski P., Król R., Nowak-Szpak A., Ozdoba M. (2022). A Preliminary Studies of the Impact of a Conveyor Belt on the Noise Emission. Sustainability.

[8] Bortnowski P., Król R., Ozdoba M. (2023). Modelling of transverse vibration of conveyor belt in aspect of the trough angle. Sci. Rep..

[9] Bortnowski P., Doroszuk B., Krol R., Marasova D., Moravic M., Ozdoba M. (2023). Forecasting blockades of conveyor transfer points based on vibrodiagnostics. Measurement.

[10] Bortnowski P., Gondek H., Król R., Marasova D., Ozdoba M. (2023). Detection of Blockages of the Belt Conveyor Transfer Point Using an RGB Camera and CNN Autoencoder. Energies.

[11] Siami M., Barszcz T., Zimroz R. (2024). Advanced Image Analytics for Mobile Robot-Based Condition Monitoring in Hazardous Environments: A Comprehensive Thermal Defect Processing Framework. Sensors.

[12] Bogacz P., Cieślik Ł., Osowski D., Kochaj P. (2022). Analysis of the Scope for Reducing the Level of Energy Consumption of Crew Transport in an Underground Mining Plant Using a Conveyor Belt System Mining Plant. Energies.

[13] Topolsky D., Topolskaya I., Plaksina I., Shaburov P., Yumagulov N., Fedorov D., Zvereva E. (2022). Development of a Mobile Robot for Mine Exploration. Processes.

[14] Siami M., Barszcz T., Wodecki J., Zimroz R., Ball A.D., Ouyang H., Sinha J.K., Wang Z. (2024). Deep Learning-Based Semantic Segmentation of Thermal Defects Using AResU-Net and REAL-ESRGAN for the Infrared Image Resolution Enhancement. Proceedings of the UNIfied Conference of DAMAS, IncoME and TEPEN Conferences (UNIfied 2023).

[15] Siami M., Barszcz T., Wodecki J., Zimroz R. (2022). Automated ir Image Segmentation for Identification of Overheated Idlers in Belt Conveyor Systems Based on Outlier-Detection Method. https://papers.ssrn.com/sol3/papers.cfm?abstract_id=4054247.

[16] Wang T., Lu G., Yan P. (2019). Multi-sensors based condition monitoring of rotary machines: An approach of multidimensional time-series analysis. Measurement.

[17] Siami M., Shiri H., Barszcz T., Wodecki J., Zimroz R. Information Fusion of Infrared Images and Acoustic Signals for Bearing Fault Diagnosis of Rotating Machinery. Proceedings of the Surveillance, Vibrations, Shock and Noise, Institut Supérieur de l′Aéronautique et de l′Espace [ISAE-SUPAERO].

[18] Polikar R. (2012). Ensemble learning. Ensemble Machine Learning: Methods and Applications.

[19] Sagi O., Rokach L. (2018). Ensemble learning: A survey. Wiley Interdiscip. Rev. Data Min. Knowl. Discov..

[20] Rokach L. (2019). Ensemble Learning: Pattern Classification Using Ensemble Methods.

[21] Kannan V., Dao D.V., Li H. (2023). An information fusion approach for increased reliability of condition monitoring with homogeneous and heterogeneous sensor systems. Struct. Health Monit..

[22] Durrant-Whyte H., Henderson T.C., Siciliano B., Khatib O. (2016). Multisensor Data Fusion. Springer Handbook of Robotics.

[23] Joyce J. (2003). Bayes’ Theorem. https://plato.stanford.edu/entries/bayes-theorem/.

[24] Matthies L., Elfes A. Integration of sonar and stereo range data using a grid-based representation. Proceedings of the 1988 IEEE International Conference on Robotics and Automation.

[25] Bell K., Corwin T., Stone L., Streit R. (2013). Bayesian Multiple Target Tracking.

[26] Babu A.C., Karri R.K., Nisha M.S. Sensor data fusion using Kalman filter. Proceedings of the 2018 International Conference on Design Innovations for 3Cs Compute Communicate Control (ICDI3C).

[27] Abdulhafiz W.A., Khamis A. Bayesian approach to multisensor data fusion with Pre-and Post-Filtering. Proceedings of the 2013 10th IEEE International Conference on Networking, Sensing and Control (ICNSC).

[28] Thrun S. (2002). Probabilistic robotics. Commun. ACM.

[29] Ganeriwal S., Balzano L.K., Srivastava M.B. (2008). Reputation-based framework for high integrity sensor networks. Acm Trans. Sens. Netw. (TOSN).

[30] Casavola A., Franzè G., Tedesco F. Sensors Selection via a Distributed Reputation Mechanism: An Information Fusion Approach. Proceedings of the 2021 26th IEEE International Conference on Emerging Technologies and Factory Automation (ETFA).

[31] Al-Maslamani N.M., Abdallah M., Ciftler B.S. (2023). Reputation-aware multi-agent DRL for secure hierarchical federated learning in IoT. IEEE Open J. Commun. Soc..

[32] Zuo Y., Liu J. (2017). A reputation-based model for mobile agent migration for information search and retrieval. Int. J. Inf. Manag..

[33] Liu C., Zhang G., Rong Y., Shao W., Meng J., Li G., Huang Y. (2023). Hybrid metric-feature mapping based on camera and Lidar sensor fusion. Measurement.

[34] Brell M., Rogass C., Segl K., Bookhagen B., Guanter L. (2016). Improving sensor fusion: A parametric method for the geometric coalignment of airborne hyperspectral and LiDAR data. IEEE Trans. Geosci. Remote Sens..

[35] Yang G.Z., Andreu-Perez J., Hu X., Thiemjarus S. (2014). Multi-sensor fusion. Body Sensor Networks.

[36] Xiao F., Wen J., Pedrycz W., Aritsugi M. (2024). Complex evidence theory for multisource data fusion. Chin. J. Inf. Fusion.

[37] Shitsukane A., Cheruiyot W., Otieno C., Mvurya M. Fuzzy logic sensor fusion for obstacle avoidance mobile robot. Proceedings of the 2018 IST-Africa Week Conference (IST-Africa).

[38] Kobayashi K., Cheok K.C., Watanabe K., Munekata F. (2002). Accurate differential global positioning system via fuzzy logic Kalman filter sensor fusion technique. IEEE Trans. Ind. Electron..

[39] Van Nam D., Gon-Woo K. (2023). Learning type-2 fuzzy logic for factor graph based-robust pose estimation with multi-sensor fusion. IEEE Trans. Intell. Transp. Syst..

[40] Wang Z., Xu X., Song D., Zheng Z., Li W. (2025). A Novel Bearing Fault Diagnosis Method Based on Improved Convolutional Neural Network and Multi-Sensor Fusion. Machines.

[41] Waske B., van der Linden S. (2008). Classifying multilevel imagery from SAR and optical sensors by decision fusion. IEEE Trans. Geosci. Remote Sens..

[42] Levitin G., Lisnianski A. (2001). Reliability optimization for weighted voting system. Reliab. Eng. Syst. Saf..

[43] Gou J., Xiong T., Kuang Y. (2011). A Novel Weighted Voting for K-Nearest Neighbor Rule. J. Comput..

[44] Mian T., Choudhary A., Fatima S. (2022). A sensor fusion based approach for bearing fault diagnosis of rotating machine. Proc. Inst. Mech. Eng. Part O J. Risk Reliab..

[45] Gültekin Ö., Çinar E., Özkan K., Yazıcı A. (2022). A novel deep learning approach for intelligent fault diagnosis applications based on time-frequency images. Neural Comput. Appl..

[46] Kou R., Lian S.w., Xie N., Lu B.e., Liu X.m. (2022). Image-based tool condition monitoring based on convolution neural network in turning process. Int. J. Adv. Manuf. Technol..

[47] Wang Z., Oates T. (2015). Imaging time-series to improve classification and imputation. arXiv.

[48] Karabacak Y.E., Gürsel Özmen N., Gümüşel L. (2020). Worm gear condition monitoring and fault detection from thermal images via deep learning method. Eksploat. I Niezawodn..

[49] Siami M., Barszcz T., Wodecki J., Zimroz R. (2022). Automated Identification of Overheated Belt Conveyor Idlers in Thermal Images with Complex Backgrounds Using Binary Classification with CNN. Sensors.

[50] Antoni J. (2007). Cyclic spectral analysis of rolling-element bearing signals: Facts and fictions. J. Sound Vib..

[51] Leevy J.L., Khoshgoftaar T.M., Bauder R.A., Seliya N. (2018). A survey on addressing high-class imbalance in big data. J. Big Data.

[52] Masko D., Hensman P. (2015). The Impact of Imbalanced Training Data for Convolutional Neural Networks. https://www.semanticscholar.org/paper/The-Impact-of-Imbalanced-Training-Data-for-Neural-Masko-Hensman/62e81797fff75603a3d7c7759e6efac4fd2b6b31.

[53] Lee H., Park M., Kim J. Plankton classification on imbalanced large scale database via convolutional neural networks with transfer learning. Proceedings of the 2016 IEEE International Conference on Image Processing (ICIP).

[54] Jumaboev S., Jurakuziev D., Lee M. (2022). Photovoltaics Plant Fault Detection Using Deep Learning Techniques. Remote Sens..

[55] Lindsay G.W. (2021). Convolutional neural networks as a model of the visual system: Past, present, and future. J. Cogn. Neurosci..

[56] LeCun Y., Bengio Y., Hinton G. (2015). Deep learning. Nature.

[57] Rezazadeh Azar E., McCabe B. (2012). Automated visual recognition of dump trucks in construction videos. J. Comput. Civ. Eng..

[58] Deng J., Dong W., Socher R., Li L.J., Li K., Fei-Fei L. Imagenet: A large-scale hierarchical image database. Proceedings of the 2009 IEEE Conference on Computer Vision and Pattern Recognition.

[59] Simonyan K., Zisserman A. (2014). Very deep convolutional networks for large-scale image recognition. arXiv.

[60] He K., Zhang X., Ren S., Sun J. Deep residual learning for image recognition. Proceedings of the IEEE Conference on Computer Vision and Pattern Recognition.

[61] Szegedy C., Liu W., Jia Y., Sermanet P., Reed S., Anguelov D., Erhan D., Vanhoucke V., Rabinovich A. Going deeper with convolutions. Proceedings of the IEEE Conference on Computer Vision and Pattern Recognition.

[62] Chollet F. Xception: Deep learning with depthwise separable convolutions. Proceedings of the IEEE Conference on Computer Vision and Pattern Recognition.

[63] Ho T.K. Random Decision Forest. Proceedings of the 3rd International Conference on Document Analysis and Recognition.

[64] Rahman M., Cao Y., Sun X., Li B., Hao Y. (2021). Deep pre-trained networks as a feature extractor with XGBoost to detect tuberculosis from chest X-ray. Comput. Electr. Eng..

[65] Tang J., Su Q., Su B., Fong S., Cao W., Gong X. (2020). Parallel ensemble learning of convolutional neural networks and local binary patterns for face recognition. Comput. Methods Programs Biomed..

[66] Sultana N., Sharma N., Sharma K.P., Verma S. (2020). A sequential ensemble model for communicable disease forecasting. Curr. Bioinform..

[67] Mohammed A., Kora R. (2023). A comprehensive review on ensemble deep learning: Opportunities and challenges. J. King Saud-Univ.-Comput. Inf. Sci..

[68] Valle C., Saravia F., Allende H., Monge R., Fernández C. (2010). Parallel approach for ensemble learning with locally coupled neural networks. Neural Process. Lett..

[69] Valdovinos R.M., Sánchez J.S., Barandela R. (2005). Dynamic and static weighting in classifier fusion. Proceedings of the Pattern Recognition and Image Analysis: Second Iberian Conference, IbPRIA 2005, Estoril, Portugal, 7–9 June 2005.

[70] Siami M., Barszcz T., Wodecki J., Zimroz R. (2022). Design of an Infrared Image Processing Pipeline for Robotic Inspection of Conveyor Systems in Opencast Mining Sites. Energies.

